# Optimization of Urea Based Protein Extraction from Formalin-Fixed Paraffin-Embedded Tissue for Shotgun Proteomics

**DOI:** 10.1155/2016/4324987

**Published:** 2016-08-31

**Authors:** Stephen A. Luebker, Scott A. Koepsell

**Affiliations:** Department of Pathology and Microbiology, University of Nebraska Medical Center, 985900 Nebraska Medical Center, Omaha, NE 68198-5900, USA

## Abstract

Urea based protein extraction of formalin-fixed paraffin-embedded (FFPE) tissue provides the most efficient workflow for proteomics due to its compatibility with liquid chromatography electrospray ionization tandem mass spectrometry (LC-ESI-MS/MS). This study optimizes the use of urea for proteomic analysis of clinical FFPE tissue. A series of protein extraction conditions manipulating temperature and buffer composition were compared to reduce carbamylation introduced by urea and increase protein detection. Each extraction was performed on a randomized pair of serial sections of homogenous FFPE tissue and analyzed with LC-ESI-MS/MS. Results were compared in terms of yield, missed cleavages, and peptide carbamylation. Lowering extraction temperature to 60°C decreased carbamylation at the cost of decreased protein detection and yield. Protein extraction for at least 20 minutes at 95°C followed by 60°C for 2 hours maximized total protein yield while maintaining protein detection and reducing carbamylation by 7.9%. When accounting for carbamylation during analysis, this modified extraction temperature provides equivalent peptide and protein detection relative to the commercially available Qproteome® FFPE Tissue Kit. No changes to buffer composition containing 7 M urea, 2 M thiourea, and 1 M ammonium bicarbonate resulted in improvements to control conditions. Optimized urea in-solution digestion provides an efficient workflow with maximized yields for proteomic analysis of clinically relevant FFPE tissue.

## 1. Introduction

We recently applied a urea in-solution digestion (UISD) method to prepare protein from FFPE tissue for liquid chromatography tandem mass spectrometry (LC-ESI-MS/MS), providing a more efficient workflow relative to Qproteome FFPE Tissue Kit [1]. UISD poses several advantages to other preparation techniques: preparation is carried out in a single tube minimizing sample handling, urea is LC-ESI-MS/MS compatible requiring no additional washing beyond desalting, and both of these factors enhance sample throughput. However, artificial carbamylation is introduced when using urea at elevated temperatures, reducing trypsin digestion efficiency. In solution, urea is in equilibrium with isocyanate and ammonium [2]. Between pH 7 and 9, isocyanate reacts with *α*-amines on N-termini and *ε*-amines on lysine side chains of peptides to form carbamyl groups [3]. Trypsin digestion efficiency was reduced by carbamylation, and UISD had more missed cleavages and a lower lysine to arginine (K : R) peptide terminal amino acid ratio relative to the commercially available Qproteome FFPE Tissue Kit (Qkit) [1]. Ammonium bicarbonate, included in the UISD extraction buffer, inhibits carbamylation when using urea as a protein denaturant, but its efficacy is greatly reduced above 37°C [4, 5]. Despite having this drawback, UISD preparation detected similar numbers of peptides and proteins obtained using Qkit and some not detected by Qkit with a bias toward arginine terminal peptides. It has been demonstrated previously that FFPE tissue shows high equivalence with fresh tissue, but FFPE preservation introduces peptide modifications, particularly lysine methylation [6]. As FFPE tissue appears to be an unreliable substrate for investigating lysine modifications, the introduction of lysine carbamylation primarily affects proteomic study by reducing cleavage efficiency. In addition, the introduction of artificial carbamylation with isotopically labeled urea can provide an alternative quantitative tool to conventional methods [7]. The benefits of UISD as a preparation method outweigh the drawback of reduced cleavage efficiency, and in this study we optimize its application to FFPE tissue.

There are multiple approaches which may enhance protein solubility and enhance digestion efficiency for UISD. In this study, we test each condition separately and some in combination to find the optimum identifiable peptide solubility while maintaining the minimum level of introduced carbamylation. Different factors including buffer composition, pH, temperature of extraction, and duration of extraction were investigated.

## 2. Materials and Methods

An anonymized FFPE melanoma tumor tissue sample was obtained with Institutional Review Board approval. The tumor was fixed using 10% neutral buffered formalin and processed by an automated processor into a paraffin-embedded block. Serial 4 *μ*m slices were made on a microtome and mounted on glass slides. The first and last slices were stained with hematoxylin and eosin to ensure the tissue was homogenous tumor cells across serial sections and to select tissue for analysis. Slide mounted FFPE tissue sections were deparaffinized twice in xylene for 5 min followed by two washes in 100% (v/v) ethanol for 5 min. The tissue was then hydrated in 85% (v/v) ethanol for 1 min, 70% (v/v) ethanol for 1 min, and distilled water for 1 min. Protein was macrodissected with a 30-gauge needle from pairs of serial sections that were randomized between each set of extraction conditions with a total area of 1650 mm^2^. Tissue was placed directly into a 2 mL microtube containing 160 *μ*L of freshly prepared 7 M urea, 2 M thiourea, and 1 M ammonium bicarbonate with modifications outlined in Table 1 [1]. Protein digestion was carried out using reconstituted proteomics grade trypsin with concentrations outlined in Table 1 (Sigma-Aldrich, St. Louis, MO, USA). After digestion, 99.8% acetic acid (Acros Organics, Fair Lawn, NJ, USA) was added dropwise to reduce to pH 4, and the solution was desalted using C18 Peptide Cleanup Tubes according to the manufacturer's instructions (Agilent Technologies Inc., Wilmington, DE, USA). Total protein was quantified by the reducing agent compatible bicinchoninic acid protein assay according to the manufacturer's instruction (Pierce, Rockford, IL, USA).

Three technical replicate injections from each extraction condition were analyzed using LC-ESI-MS/MS system in a nanospray configuration (ABSciex 6600 TripleTOF®, AB SCIEX, Framingham, MA, USA) coupled with Eksigent NanoLC 415 with a cHiPLC system (Eksigent, Dublin, CA, USA) as previously described [1]. The Paragon*™* and Pro Group*™* algorithms were used for protein identification and grouping in ProteinPilot 5.0 (AB SCIEX, Framingham, MA, USA). Data analysis parameters included data-dependent analysis,* H. sapiens* as the species, iodoacetamide cysteine alkylation, and trypsin digestion, and the analysis was carried out both with and without urea denaturation considered as a special factor. All data files were searched using a UniProtKB/SwissProt database downloaded in August of 2015. Positive protein detection for comparisons between methods was defined as two peptide matches per protein and a global false discovery rate of 1%. Positive peptide detection was limited to a global false discovery rate of 1%.

## 3. Results and Discussion

A series of conditions were tested to inhibit peptide carbamylation and to maximize protein yield (Table 1). Peptide carbamylation was an important factor for the performance of UISD that can affect spectral data analysis. A summary of results from ProteinPilot without urea denaturation considered as a special factor for data analysis is shown in Table 2 and the summary of results from ProteinPilot with urea denaturation considered as a special factor is shown in Table 3. The improvements with this search setting are demonstrated by the gain in spectra and peptides detected at <1% FDR. Improvements in the identification of peptide spectra indicate better detection of peptides with urea denaturation induced artifacts. All UISD conditions increased the number of spectra, peptides, and proteins detected with urea denaturation considered as a special factor (Tables 2 and 3). Additionally, the number of carbamyl groups detected and the number of missed cleavages detected both increased for all UISD conditions under the same search parameters (Tables 2 and 3). The most significant improvement for UISD is that protein and peptide detection were better than Qkit for multiple conditions tested when accounting for urea induced modifications, and the values in Table 3 are used in further comparisons between methods.

The Paragon algorithm is used in ProteinPilot to identify peptides from mass spectra measured during LC-ESI-MS/MS. Shilov et al. give a complete description on how the Paragon algorithm uses a scoring method to modulate search space based on evidence from sequence tags [8]. Briefly, probabilities are calculated for peptide hypotheses based on predefined parameters set by the user such as digestion enzyme, alkylating agent, and special factors; these probabilities are empirically determined by measuring how frequently they occur [8]. This is an important feature of the algorithm for this study because the special factor of urea denaturation selected in the search settings provided better peptide detection for UISD preparation of FFPE tissue. Selecting urea denaturation accounts for the empirically determined frequency that urea induced modifications like carbamylation occur which are built into the algorithm to influence the probability of a given peptide hypothesis. In conjunction with peptide hypothesis probabilities, segment probabilities based on* de novo* sequence tags termed “Sequence Temperature Value” for regions of the sequence database and protein probabilities based on initial precursor mass filtered search are used to generate an overall probability that determines the search space segments of the sequence database [8].

Extraction temperature affects the total protein yield from FFPE tissue, but greater overall protein yield did not always result in the most detected proteins. The control condition with longest high temperature extraction yielded only 2.9 *μ*g/mm^3^ but detected the most proteins of all UISD conditions (Table 3). Ikeda et al. and Fowler et al. found short term intense heating at 100°C for 20 minutes followed by extended mild heating at 60°C or 80°C for 2 hours provided the optimum protein recovery from FFPE tissue [9, 10]. Similar conditions were applied to UISD starting with extraction at 60°C for 2 hours in condition (A) as shown in Table 1, and it yielded 1.1 *μ*g/mm^3^ more than control. Even though condition (A) had greater overall protein yield, 347 fewer proteins were detected relative to control (Table 3). Extracting protein for 5 minutes at 95°C prior to 2 hours at 60°C provided no change to results relative to condition (A) (data not shown). Increasing extraction time at 95°C to 20 minutes prior to extraction at 60°C for 2 hours in condition (B) increased the total protein yield by 3.3 *μ*g/mm^3^ relative to control. Longer extraction time at mild temperature used in condition (B) resulted in detection of only 16 fewer proteins than control while increasing the number of detected peptides by 1030 (Table 3). In condition (C), extraction at 95°C was maintained at 20 minutes, but it was followed by 80°C instead of 60°C for 2 hours (Table 1). Condition (C) yielded 2.3 *μ*g/mm^3^ more protein than control but also resulted in detection of 44 fewer proteins.

Since the extraction temperature in condition (B) made the most improvement in peptide detection, the remaining conditions were tested using these extraction temperatures combined with changes to buffer composition (Table 1). In an attempt to reduce isocyanate formation, 3 M ammonium bicarbonate (Sigma-Aldrich, St. Louis, MO, USA) alone was introduced to the extraction buffer, but carbamylation remained unchanged and fewer proteins were detected than control (data not shown). In condition (D), ammonium bicarbonate was increased to 2 M and pH was reduced to 7.2 with HCl (Sigma-Aldrich, St. Louis, MO, USA) to test the effect of pH on extraction while maintaining a higher concentration of ammonium. Total protein yield for condition (D) was 1.6 *μ*g/mm^3^ lower than condition (B), and it detected 384 fewer proteins and 2324 fewer peptides. One major drawback for using neutral pH to enhance digestion efficiency is that extraction buffers with pH > 8 have been shown previously to be important for protein yield [11].

Other buffer changes were made to maximize solubility and protein yield beyond gains seen with temperature manipulation. In order to approach protein yield of Qkit, 6%  *β*-mercaptoethanol (Sigma-Aldrich, St. Louis, MO, USA) was added for extraction under reducing conditions, but yield and protein detection were reduced relative to control (data not shown). Shen et al. found that 0.2% Zwittergent 3-16 yielded better results than buffer containing 8 M urea, and the result was attributed to the enhanced lytic strength of the detergent [12]. In order to enhance cell lysis and protein solubility, 0.2% Zwittergent 3-16 (Santa Cruz Biotechnology, Dallas, TX, USA) was included in the extraction buffer for condition (E), and this was combined with the extraction temperatures from condition (B) (Table 1). The addition of 0.2% Zwittergent 3-16 decreased total protein yield 1.19 *μ*g/mm^3^ relative to condition (B), and 930 fewer peptides and 55 fewer proteins were detected (Table 3). Due to the limited amount of available clinical tissue that was used for this study, only a single tissue section was available to test the effect of Zwittergent 3-16 on total protein yield. As the total yield for condition (E), 6.01 *μ*g/mm^3^, was only slightly lower than the 6.16 *μ*g/mm^3^ yield for condition (B) and protein and peptide detection levels were within previously seen ranges, a second replicate was not included for this modification. In addition, the number of proteins and peptides detected was not sufficiently different from condition (B) to justify a second tissue section.

After maximizing the peptide detection and total protein yield of UISD, the second goal for manipulating extraction temperature was to limit lysine carbamylation for enhanced digestion efficiency. The percentage of carbamylated peptides reached a minimum of 2.2% for UISD with only 60°C extraction in condition (A), and it also resulted in K : R of 0.643, the highest obtained using UISD. Condition (B) had 7.9% less total carbamylation than control while detecting 1030 more peptides. The percentage of missed cleavages out of the total number of peptides was 7.3% lower and total carbamylation was 7.9% lower for condition (B) relative to control. Condition (C) reduced carbamylation by only 3.1% relative to control.

As an alternative to decrease lysine carbamylation, reduction of pH to near 7 should increase bias toward N-terminal carbamylation rather than lysine carbamylation since *α*-amines react faster at more neutral pH than *ε*-amines [3]. Condition (D) reduced pH to 7.2 with 2 M ammonium bicarbonate and successfully decreased total carbamylation to 17.7% compared to 52.4% for condition (B) using the same extraction temperature. The fraction of carbamyl lysine residues out of total peptides was reduced by 23.8% relative to condition (B), and carbamyl N-termini only decreased by 0.2%. Missed cleavages for condition (D) were also decreased by 29.3% relative to condition (B). The reduction in carbamylation for condition (D) approached condition (A) by lowering pH instead of temperature, but peptide and protein detection was decreased for both relative to condition (B) or control (Table 3). Both condition (A) and condition (D) had reduced carbamylation and showed smaller gains in terms of the number of proteins, peptides, and spectra gained when accounting for urea denaturation in the search conditions or not, further demonstrating the importance of accounting for urea induced modifications (Tables 2 and 3).

Elimination of high temperature extraction and neutral buffer pH both reduced carbamylation only at the cost of reduced overall protein and peptide detection. The sample is also heated at 37°C for overnight digestion, which is a long period to introduce additional peptide carbamylation at a slower rate. As an alternative to extraction manipulation, trypsin digestion was limited to 30 minutes at 50°C for condition (F) using extraction time and temperature from condition (B) in order to reduce overall sample exposure time to heating while maintaining adequate yields (Table 1). Condition (F) detected 192 fewer proteins and 1163 fewer peptides than control. Carbamylation for condition (F) was increased by 1.0% relative to condition (B). Missed cleavages were elevated for condition (F) relative to all other UISD conditions, and arginine missed cleavages in particular were elevated for condition (F), indicating reduced overall digestion efficiency for rapid digestion independent of lysine carbamylation (Table 3). Any enhancement cleavage efficiency at lysine residues was negated by reduced overall digestion efficiency under rapid digestion. Accelerated digestion reduces sequence coverage and has been cautioned against for complex protein mixtures [13]. As previously mentioned for condition (E), only a limited number of tissue sections were available for this study. A single tissue section replicate was used for condition (F) due to the clearly apparent reduction in digestion efficiency from the increased number of missed arginine cleavages from a single tissue section. In this study, overall digestion efficiency was reduced, but accelerated preparation may outweigh the reduced efficiency given the need for rapid sample preparation.

**Figure 1:**
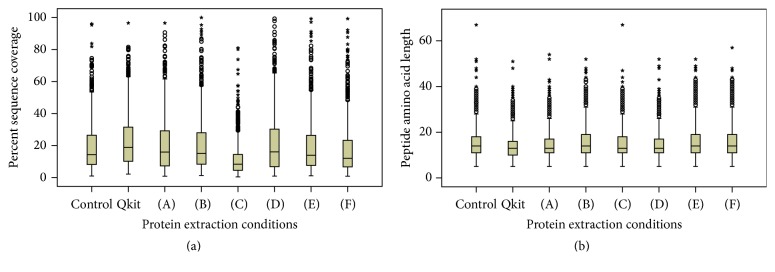
Side-by-side boxplots of detected protein percent sequence coverage (a) and peptide amino acid length (b) between each set of extraction conditions. Protein sequence coverage at the 95% confidence level and peptide lengths were detected by analysis in ProteinPilot*™* using urea denaturation as a special factor.

UISD provides a protein extraction workflow from FFPE tissue with minimal sample handling steps, but it introduces significant artificial modifications to peptides in the form of carbamylation. Carbamylation reduces trypsin digestion at lysine residues leading to the generation of predominantly arginine terminal peptides. Despite this bias, distribution of sequence coverage across the set of conditions is very similar to those obtained using Qkit with positive skew across all conditions (Figure 1(a)). The higher temperatures used in control and condition (C) both show a smaller interquartile range in addition to reduced median sequence coverage relative to all other conditions (Table 3). Hildonen et al. demonstrated that incomplete trypsin digestion provides more complete proteome coverage in a simple protein mixture by minimizing small peptide generation and decreased ion intensity [14]. The distribution of detected peptide lengths across the different conditions varied little (Figure 1(b)). UISD condition (B) had a slightly higher average peptide length of 15 ± 6 amino acids compared to Qkit with 13 ± 5. No advantage in sequence coverage was seen relative to Qkit in terms of overall distribution of protein sequence coverage, but conditions with the optimum temperature (B), (D), (E), and (F) all show greater numbers of proteins with very high sequence coverage as outliers in the distribution (Figure 1(a)). The lack of any improvement may be due to limitations of the mass detection range by LC-ESI-MS/MS for longer peptides or limited sampling due to the relatively short 60-minute elution gradient used for LC-ESI-MS/MS (Figure 1(a)). Improvements to sequence coverage and overall detection of peptides can be made by peptide fractionation prior to LC-ESI-MS/MS. As we pointed out in our previous paper analyzing this method, overall protein detection and total protein yield vary across studies using different instrumentation, tissue type, and data analysis methods, making direct comparisons difficult across studies [1]. When artifacts introduced by UISD using control conditions are accounted for during analysis, the optimized UISD condition (B) detects more proteins and peptides than Qkit with comparable distribution of sequence coverage and equivalent yield.

## 4. Conclusion

Protein extraction at 95°C for 20 minutes followed by 60°C for 2 hours provided the best total protein yield and best peptide detection for the tissue analyzed. Modifications to extraction buffer or digestion conditions all reduced overall performance for UISD. Despite artificially introducing carbamylation, the optimized UISD conditions detected more peptides and proteins relative to Qkit with a comparable distribution of protein sequence coverage making it a useful method for proteomic studies. During data analysis of mass spectra, considering urea denaturation as a special factor is important for maximizing protein and peptide detection with the Paragon algorithm. This study has two main limitations: first using only a limited amount of clinically available tissue for extraction method comparisons and second the inability to modify or view specific calculations in the proprietary software used to analyze mass spectra. UISD provides efficient protein extraction that can be applied to clinically relevant FFPE tissue for proteomic analysis providing a useful tool for target development in immunohistochemical studies.

## Figures and Tables

**Table 1:** Summary of protein extraction conditions. Yield was calculated as the total *µ*g of protein divided by the area of the tissue section multiplied by the section thickness of 4 *µ*m.

	Control	(A)	(B)	(C)	(D)	(E)	(F)	Qkit
Extraction buffer	7 M urea 2 M thiourea 1 M AmBic	Same as control			7 M urea 2 M thiourea 2 M AmBic pH 7.2	7 M urea 2 M thiourea 1 M AmBic 0.2% Zwittergent 3-16	Same as control	EXB plus extraction buffer

First temperature	95°C 1.5 hr	N/A	95°C 20 min					100°C 20 min

Second temperature	N/A	60°C 2 hr		80°C 2 hr	60°C 2 hr			80°C 2 hr

Trypsin digestion	Overnight 37°C 1 : 20 (w/w)						30 min 50°C 1 : 5 (w/w)	Overnight 37°C 1 : 20 (w/w)

Total protein (*µ*g)	19.4	26.3	40.6	34.4	30.6	19.9^*∗*^	16.4^*∗*^	41.0

Protein yield (*µ*g/mm^3^)	2.94	3.99	6.16	5.22	4.63	6.03	4.97	6.22

^*∗*^Protein extracted from a single section of tissue.

**Table 2:** Summary of LC-ESI-MS/MS data without considering urea denaturation as special factor in ProteinPilot. Detected peptides were limited to 1% global FDR. Detected proteins were limited to 2 peptides and 1% global FDR. Percent total carbamyl lysine was calculated as the number of total carbamyl groups out of the total number of peptides. Percent missed cleavages were calculated as the total number of missed cleavages out of the total number of peptides.

	Control	Qkit	(A)	(B)	(C)	(D)	(E)	(F)
Detected spectra	43657	67411	45073	49333	40871	45571	52207	35281
Detected proteins	948	1141	891	1027	888	835	957	836
Detected peptides	6318	9628	7104	7780	5868	7292	7153	6132
K-terminal peptides	180	4394	2725	734	124	2552	411	301
R-terminal peptides	6030	4986	4234	6936	5646	4627	6638	5718
Peptide K : R	0.03	0.881	0.644	0.106	0.022	0.552	0.05	0.053
Carbamyl N-terminus	506	12	36	564	451	241	468	420
Carbamyl lysine	1211	2	33	1467	1066	428	1120	959
Total carbamyl groups	1749	30	72	2057	1535	681	1612	1407
Percent total carbamylation	27.7	0.3	1.0	26.4	26.2	9.3	22.5	22.9
Total missed cleavages	3052	1004	2493	3728	2656	2517	3749	3833
Missed lysine cleavages	2322	781	1811	3021	2077	1984	2796	2529
Missed arginine cleavages	730	223	682	707	579	533	953	1304
Percent missed cleavages out of total peptides	48.3	10.4	35.1	47.9	45.3	34.5	52.4	62.5

**Table 3:** Summary of LC-ESI-MS/MS data when considering urea denaturation as a special factor in ProteinPilot. Detected peptides were limited to 1% global FDR. Detected proteins were limited to 2 peptides and 1% global FDR. Percent total carbamyl lysine was calculated as the number of total carbamyl groups out of the total number of peptides. Percent missed cleavages were calculated as the total number of missed cleavages out of the total number of peptides.

Extraction	Control	Qkit	(A)	(B)	(C)	(D)	(E)	(F)
Detected spectra	63337	67411	46279	64889	59074	49213	68932	48689
Detected proteins	1252	1141	905	1236	1208	852	1181	1060
Median percent sequence coverage	11.4	15.2	15.5	11.9	10.6	16.0	10.9	10.7
Percent sequence coverage interquartile range	15.9	21.1	21.0	17.5	14.8	23.7	16.3	15.7
Detected peptides	9025	9628	7596	10055	8431	7731	9125	7862
Mean peptide length	15 ± 6	13 ± 5	14 ± 5	15 ± 6	15 ± 6	14 ± 5	15 ± 6	16 ± 6
K-terminal peptides	244	4394	2911	966	209	2739	513	369
R-terminal peptides	8624	4986	4530	8936	8074	4875	8474	7343
Peptide K : R	0.028	0.881	0.643	0.108	0.026	0.562	0.061	0.050
Carbamyl N-terminus	283	12	76	200	200	136	180	194
Carbamyl lysine	3722	2	89	3866	3466	1127	3427	3009
Total carbamyl groups	5438	30	170	5268	4821	1368	4727	4196
Percent total carbamylation	60.3	0.02	2.2	52.4	57.2	17.7	51.8	53.4
Total missed cleavages	6795	1004	2747	6835	5950	2994	6485	6367
Missed lysine cleavages	5705	781	2000	5908	5106	2438	5265	4717
Missed arginine cleavages	1090	223	747	927	844	556	1220	1650
Percent missed cleavages out of total peptides	75.3	8.1	36.1	68.0	70.6	38.7	71.1	81.0
